# Obesity at age 20 and the risk of miscarriages, irregular periods and reported problems of becoming pregnant: the Adventist Health Study-2

**DOI:** 10.1007/s10654-012-9749-8

**Published:** 2012-12-08

**Authors:** Bjarne K. Jacobsen, Synnøve F. Knutsen, Keiji Oda, Gary E. Fraser

**Affiliations:** 1 Department of Community Medicine, University of Tromsø, 9037 Tromsø, Norway; 2 Department of Epidemiology, Biostatistics and Population Medicine, School of Public Health, Loma Linda University, Loma Linda, CA 92350 USA

**Keywords:** Obesity, Menstruation, Abortion, spontaneous, Fertility, Seventh-day adventist

## Abstract

In a group of 46,000 North-American Adventist women aged 40 and above, we investigated the relationships between body mass index (BMI, kg/m^2^) at age 20 and the proportion of women who reported at least one miscarriage, periods with irregular menstruation or failing to become pregnant even if trying for more than one straight year. Approximately 31, 14 and 17 %, respectively, reported the three different problems related to reproduction. Positive age- and marital status adjusted relationships were found between BMI at age 20 and periods with irregular menstruation or failing to become pregnant even if trying for more than 1 year, but not with the risk of miscarriages. Women with BMI ≥ 32.5 kg/m^2^ when aged 20 had approximately 2.0 (95 % CI: 1.6, 2.4) and 1.5 (95 % CI: 1.3, 1.9) higher odds for irregular periods or failing to get pregnant, respectively, than women with BMI in the 20–24.9 kg/m^2^ bracket. These relationships were consistently found in a number of strata of the population, including the large proportion of the women who never had smoked or never used alcohol. Underweight (BMI < 18.5 kg/m^2^) when aged 20 marginally (approximately 15 %) increased the risk of failing to get pregnant within a year. Thus, obesity at age 20 increases the risk of reporting some specific reproductive problems, but not the risk of miscarriages.

**Electronic supplementary material:**

The online version of this article (doi:10.1007/s10654-012-9749-8) contains supplementary material, which is available to authorized users.

## Introduction

Pregnancy-related problems are only some of the many adverse effects of obesity. Complications may occur during pregnancy (e.g. hypertension, thromboembolism, diabetes) and during labor (e.g. fetal distress, dystocia and instrumental delivery/cesarean section). But obesity may also be related to fertility and miscarriages [1–3]. It is well known that obesity reduces the likelihood of a successful result of assisted reproduction (ART) [3, 4]. Less is known from population-based studies, however.

Miscarriages, particularly early in the pregnancy, are frequent [5], but the relationship between BMI and the risk of miscarriages among women in the general population is not established as both underweight and obesity have been reported to increase the risk of miscarriages [6–12].

Brewer and Balen [3] have recently reviewed how obesity affects adversely both conception and implantation. Time to pregnancy is longer and fecundity lower in obese women than in women at optimal weight [13–16]. Some previous studies has indicated that obesity as young women is associated with ovulatory infertility [17] and menstrual problems later in life [18].

However, other lifestyle factors are also of importance with regard to childbearing. Both smoking [19] and alcohol consumption [20] are dangerous for the fetus. Also caffeine containing beverages may be a risk factor, although the evidence is weak [21]. Such life style factors may confound or modify the relationship between body mass and reproductive health [22].

The aim of this study was to investigate, in a population of 46,000 American women aged 40 and above, how BMI (both low and high) at age 20 influences the frequency of reporting miscarriages, irregular periods or failing to become pregnant even if trying to get pregnant for one straight year or more.

The large number of women included facilitates the investigation of effects of underweight (body mass index < 18.5 kg/m^2^) as well as obesity (both body mass index 30–32.4 kg/m^2^ and body mass index ≥32.5 kg/m^2^). The women were members of the Adventist church, thus a large proportion had never (thus also during the childbearing years) smoked or used alcohol. Furthermore, the consumption of caffeine containing beverages (coffee or soft drinks) was low with approximately two-thirds never consuming this or using it less frequently than once a month.

## Materials and methods

Adventist church members living in the USA and Canada, aged 30 years and more, were included in the Adventist Health Study-2 (AHS-2) [23]. The enrolment commenced in February 2002, and concluded in December 2007. More than 96,000 participants completed the lifestyle questionnaire which took 1–3 h to complete. About 25,500 were black Adventists of US and Caribbean descent and 62,500 were females. The Adventists church encourages a healthy life style with no smoking and alcohol consumption and advises members to follow a vegetarian diet.

The comprehensive self-administered questionnaire included sections for medical history, diet, physical activity, supplement use and vegetarian food consumption. Information on marital status, ethnic group and lifestyle variables like smoking and the use of alcohol and caffeine containing beverages were available. Only 0.3 % of the women reported living in a common law marriage, and these women were in the stratified analyses included in the group of ever married women.

The female history section included information about, among other topics, menarche, irregular menstruation and difficulties in becoming pregnant (at different points during the life of the women) and the outcome of the pregnancies (including miscarriages/stillbirths, ectopic pregnancies, elective abortion and live births), and the use of oral contraceptives.

There were also simple questions about current weight and height as well as weight when aged 20. Body mass index (BMI) was computed as weight in kilograms divided by the square of height in meters (kg/m^2^).

The three dependent variables considered in our study were ever having experienced a miscarriage, menstrual irregularities and failing to become pregnant even if trying for one straight year. The women were asked to state the number of miscarriages or stillbirths she had experienced. In the main analyses, we dichotomized this information into ever/never having experienced this pregnancy outcome. Menstrual irregularities was considered present if the women answered “Yes” to the question “Have your periods ever had much reduced flow, become irregular or stopped completely for at least 6 months? Do not count during or after menopause, or when you were pregnant, or nursing a child”. If the women indicated that this happened before the age of 20 only, we did not include her in the group of women with menstrual irregularities. The women answered another question about problems with becoming pregnant: “Did you ever try for one straight year or more to become pregnant and, during that time, not become pregnant?” If the women indicated that this happened before the age of 20 only or that the only reason for the problem was that the husband had fertility problem, we did not include her in the group of women with problems becoming pregnant.

The independent variable was BMI at age 20. BMI was categorized into 6 groups: BMI < 18.5, 18.5 ≤ BMI < 20, 20 ≤ BMI < 25, 25 ≤ BMI < 30, 30 ≤ BMI < 32.5 and BMI ≥ 32.5. These groups are in accordance with the main groups recommended by the WHO for classification of underweight, normal weight, overweight and obesity (<18.5, 18.5–24.9, 25–29.9, ≥30), but the present classification is more detailed.

The present analyses were limited to 54,369 women who were between the age of 40 and 99 at enrolment. The following missing data led to exclusions: ever having been pregnant was missing for 183 women, information regarding marital status, a key determinant of childbearing, was missing for 1,102 women; an additional 4,727 women had missing information regarding BMI at age 20. We also excluded 808 women with estimated BMI lower than 16.0 kg/m^2^ or higher than 60.0 kg/m^2^ as these were considered to either reflect incorrect self-reported data concerning weight or height or severe illness. In some situations (2–4 % of the women), the information from the women was missing with regard to the three dependent variables. Thus, the number of women included in the analyses varied between 45,701 regarding information concerning irregular periods to 46,582 for information on having tried for one straight year or more to become pregnant but not having become pregnant.

In addition to age when completing the questionnaire (5 year age groups) and marital status (7 groups), the following variables were considered as possible confounders of the relationship between BMI at age 20 and the three different indicators of fertility problems: Ethnic group (blacks vs. other), level of education, age at menarche, extended use of oral contraceptives (here defined as having used oral contraceptives for 7 or more years both when aged 20–29 and when aged 30–39), parity, ever smoked and ever regularly used alcohol as well as monthly or more frequently use of caffeine containing beverages.

The statistical analyses included simple cross-tabulations, analyses of variance and multiple logistic regression analyses. Stratified analyses were conducted in order to control for confounding and evaluating possible effect modification. *p* values < 0.05 were considered statistically significant. The *p* values in the tables test the hypothesis of any difference according to BMI (in 6 categories) rather than a linear trend over BMI categories. In some situations, we also tested for a U-formed relationship, including also a quadratic term in the model. Analyses were performed using SAS Software [24].

## Results

The mean age (standard deviation) of the women at enrollment was 59.9 (14.7). Overall, 30.6, 14.1 and 16.5 % reported miscarriages, irregular periods, and problems becoming pregnant, respectively.

Table 1 displays the associations between BMI at age 20 and some relevant variables which may be associated with BMI. Women who were obese at age 20 were more likely to be relatively younger when completing the lifestyle questionnaire, never to have been married, to have relatively low education, to be black, and to have early menarche and have relatively low parity. Only 1 and 6 %, respectively, of the women were current users of tobacco or alcohol. Thus, these groups of current stimulant users were merges with former users. Monthly use of caffeinated beverages as well as ever use of tobacco or alcohol was associated with obesity.

**Table 1:** Unadjusted relationships between body mass at age 20 and demographic, reproductive and life style variables. Mean values (SD) or percentages

	Body mass index (kg/m^2^) at age 20							
	N	<18.5	18.5–19.9	20–24.9	25–29.9	30–32.4	≥32.5	*p* value
Number of women	47,549	6,815 (14.3)	10,213 (21.5)	25,866 (54.4)	3,539 (7.4)	506 (1.1)	610 (1.3)	
Age at enrolment	47,549	58.5 (11.9)	59.2 (12.3)	60.8 (12.9)	59.4 (13.3)	57.0 (12.8)	54.8 (11.2)	<0.0001
% Ever married	47,549	94.6	95.6	94.8	90.6	87.7	82.3	<0.0001
% With college degree	47,122	33.1	34.7	32.5	28.5	23.5	25.5	<0.0001
% Blacks	47,002	30.4	24.2	23.4	30.4	33.8	35.9	<0.0001
Age at menarche	47,227	12.9 (1.6)	12.7 (1.6)	12.5 (1.5)	12.2 (1.6)	12.1 (1.7)	11.7 (1.6)	<0.0001
Live births	46,334	2.3 (1.7)	2.3 (1.6)	2.4 (1.7)	2.3 (1.8)	2.1 (1.8)	1.8 (1.9)	<0.0001
% Extended OC use^a^	46,972	2.1	2.0	1.8	1.7	2.6	2.0	0.33
% Who consume caffeinated drinks	44,912	32.7	35.7	35.6	38.5	44.2	42.0	<0.0001
% Ever smoked	47,255	17.0	16.7	16.4	19.5	27.7	31.5	<0.0001
% Ever used alcohol	47,161	36.0	37.3	36.0	40.6	49.1	51.5	<0.0001

^a^Used oral contraceptives (OC) for 7 or more years both when aged 20–29 and when aged 30–39

After adjustments for age and marital status, those reporting ever to have experienced a miscarriage had increased odds of failing to become pregnant even if trying for one straight year; odds ratio (OR) 1.51 (95 % CI: 1.43–1.59). Increasing number of miscarriages (1, 2 and >2) was linearly related to the odds of reporting failing to become pregnant (OR = 1.35 (95 % CI: 1.27–1.44), 1.63 (95 % CI: 1.49–1.79) and 2.37 (95 % CI: 2.12–2.64)), respectively compared to the risk in women with no miscarriages). Also ever experienced irregular periods was positively related to failing to become pregnant even if trying for one straight year; OR = 1.72 (95 % CI: 1.61–1.83), but there was very little relationship between the experience of a miscarriage and the likelihood of reporting irregular periods (results not shown in tables).

Two percent of the women reported at least one ectopic pregnancy. Underweight or obesity at age 20 did not have any bearing on the risk of this pregnancy outcome (*p* = 0.16). When adjusted for age when completing the questionnaire and marital status, the odds for a hysterectomy before the age of 40 (15 % of the women indicated this) was approximately 35 % higher in obese women than in women with normal weight (*p* = 0.003), but after additional adjustments, for education and ethnic group, this relationship was no longer statistically significant (*p* = 0.06) (results not shown in the table).

Table 2 gives the relationships between BMI at age 20 and the likelihood of reporting at least one miscarriage, irregular periods or failing to become pregnant even if trying for one straight year. The first line represents results of analyses are adjusted for age when completing the questionnaire and marital status, the next two lines when adjusted for an increasing number of possible confounders.

**Table 2:** Relationships between body mass index at age 20 and the likelihood of having experienced a miscarriage, irregular periods or failing to become pregnant even if trying for one straight year

	Body mass index (kg/m^2^)							
	N	<18.5	18.5–19.9	20–24.9	25–29.9	30–32.4	≥32.5	*p* value
Dependent variable								
Ever experienced miscarriage								
Miscarriage	46,334	1.06 (1.00, 1.12)	1.02 (0.97, 1.07)	1.00	1.11 (1.03, 1.20)	1.11 (0.92, 1.35)	0.97 (0.80, 1.16)	0.06
Miscarriage^a^	44,975	1.04 (0.98, 1.10)	1.02 (0.97, 1.07)	1.00	1.07 (0.99, 1.16)	1.04 (0.85, 1.27)	0.92 (0.76, 1.11)	0.46
Miscarriage^b^	42,979	1.04 (0.98, 1.11)	1.02 (0.97, 1.08)	1.00	1.05 (0.97, 1.14)	1.01 (0.82, 1.24)	0.87 (0.72, 1.06)	0.40
Menstrual irregularities								
Menstrual irregularities	45,701	1.03 (0.95, 1.12)	0.98 (0.91, 1.04)	1.00	1.18 (1.07, 1.31)	1.89 (1.53, 2.33)	1.98 (1.64, 2.39)	<0.0001
Menstrual irregularities^a^	44,429	1.03 (0.95, 1.12)	0.97 (0.90, 1.04)	1.00	1.16 (1.05, 1.28)	1.84 (1.49, 2.28)	1.91 (1.58, 2.31)	<0.0001
Menstrual irregularities^c^	42,979	1.04 (0.96, 1.13)	0.97 (0.90, 1.04)	1.00	1.16 (1.04, 1.29)	1.79 (1.44, 2.23)	1.87 (1.54, 2.27)	<0.0001
Problems with becoming pregnant								
Problems with becoming pregnant	46,582	1.16 (1.08, 1.25)	1.13 (1.06, 1.20)	1.00	1.11 (1.01, 1.22)	1.46 (1.17, 1.82)	1.55 (1.26, 1.90)	<0.0001
Problems with becoming pregnant^a^	45,246	1.14 (1.06, 1.23)	1.12 (1.05, 1.20)	1.00	1.11 (1.00, 1.22)	1.49 (1.19, 1.87)	1.53 (1.25, 1.89)	<0.0001
Problems with becoming pregnant^d^	42,979	1.13 (1.04, 1.22)	1.13 (1.06, 1.21)	1.00	1.07 (0.97, 1.19)	1.36 (1.08, 1.72)	1.36 (1.10, 1.69)	<0.0001

Odds ratio (95 % CI). Adjusted for age when filling in the questionnaire and marital status

^a^Also adjusted for ever smoking, ever use of alcohol, ethnic background (blacks vs. other) and level of education

^b^Also adjusted for ever smoking, ever use of alcohol, ethnic background (blacks vs. other), level of education as well as problems with becoming pregnant, menstrual irregularities and age at menarche

^c^Also adjusted for ever smoking, ever use of alcohol, ethnic background (blacks vs. other), level of education as well as experienced a miscarriage, problems with becoming pregnant and age at menarche

^d^Also adjusted for ever smoking, ever use of alcohol, ethnic background (blacks vs. other), level of education as well as experienced a miscarriage, menstrual irregularities and age at menarche

No relationship was found for the risk of any miscarriage. Furthermore, we found no linear relationship between body mass index at age 20 and the number of miscarriages (not shown in the table). Women with BMI ≥ 32.5 kg/m^2^ at age 20 had approximately 2.0 and 1.5 higher odds for irregular menstruation or failing to get pregnant, respectively, than women with BMI in the 20–24.9 kg/m^2^ bracket. Underweight (BMI < 18.5 kg/m^2^) when aged 20 marginally (approximately 15 %) increased the risk of failing to get pregnant within a year (*p* value for quadratic term <0.001). Also for irregular menstruation, a U-formed relationship was statistically significant (*p* < 0.001), but the increased risk associated with underweight was negligible.

Adjustments for ever smoking, ever use of alcohol, ethnic background (blacks vs. other) and education in addition to age and marital status had little impact on these relationships (Table 2). Further adjustments for age at menarche as well ever experienced one of the two other dependent variables included in our analyses did not explain the statistically significantly increased risk associated with obesity. However, the increased risk of failing to become pregnant associated with both underweight and obesity was attenuated with the last set of adjustments (Table 2), but even fully adjusted (line 3), there was a statistically significant (*p* < 0.001) U-formed relationship between body mass index at age 20 and problems of becoming pregnant.

It may, however, be debatable whether it is correct to adjust for miscarriages and irregular periods when assessing the relationship between body mass index at age 20 and failing to become pregnant, as these variables may be considered intermediary. Thus, the impact of these two variables on the relationship with failing to become pregnant was assessed in a separate analysis. When adjusted for irregular periods and miscarriages in addition to smoking, alcohol, ethnic background, education and age at menarche (including 42,979 women in both analyses), the odds ratio associated with obesity (BMI ≥ 30 kg/m^2^) was attenuated from 1.45 to 1.36. The slightly increased risk associated with underweight was not influenced by this adjustment.

A number of supplementary analyses were conducted in strata of analytical population. Some of the results regarding failing to become pregnant are presented in Table 3. As evident, the displayed relationship did not depend on the age when completing the questionnaire (*p* value for interaction = 0.9), or whether the women had ever been married (*p* value for interaction = 0.6). The relationship may be somewhat weaker in blacks than in other ethnic groups (*p* value for interaction = 0.03).

**Table 3:** Stratified analyses of the relationships between body mass index at age 20 and having experienced problems getting pregnant

	Body mass index (kg/m^2^) at age 20							
	N	<18.5	18.5–19.9	20–24.9	25–29.9	30–32.4	≥32.5	*p* value
All women	46,582	1.16 (1.08, 1.25)	1.13 (1.06, 1.20)	1.00	1.11 (1.01, 1.22)	1.46 (1.17, 1.82)	1.55 (1.26, 1.90)	<0.0001
Age (years)								
40–54	18,514	1.21 (1.09, 1.35)	1.08 (0.98, 1.19)	1.00	1.23 (1.07, 1.42)	1.56 (1.15, 2.12)	1.55 (1.18, 2.04)	<0.0001
55–69	16,795	1.11 (0.98, 1.25)	1.10 (1.00, 1.23)	1.00	0.95 (0.79, 1.13)	1.42 (0.96, 2.10)	1.60 (1.12, 2.28)	0.014
70+	11,273	1.15 (0.97, 1.35)	1.28 (1.13, 1.46)	1.00	1.10 (0.90, 1.34)	1.26 (0.73, 2.17)	1.31 (0.72, 2.41)	0.008
Married								
Never	2,607	1.31 (0.81, 2.12)	0.76 (0.45, 1.30)	1.00	1.27 (0.77, 2.09)	1.49 (0.58, 3.85)	1.64 (0.81, 3.31)	0.33
Ever	43,975	1.16 (1.08, 1.25)	1.14 (1.07, 1.21)	1.00	1.10 (1.00, 1.21)	1.45 (1.16, 1.83)	1.53 (1.23, 1.89)	<0.0001
Ethnic group								
Blacks	11,583	1.12 (0.97, 1.28)	0.97 (0.85, 1.10)	1.00	1.22 (1.03, 1.46)	1.43 (0.97, 2.11)	1.29 (0.90, 1.85)	0.03
Other	34,489	1.17 (1.07, 1.27)	1.19 (1.11, 1.27)	1.00	1.07 (0.95, 1.20)	1.47 (1.12, 1.94)	1.69 (1.31, 2.17)	<0.0001
Parity								
0	7,318	1.33 (1.13, 1.55)	1.11 (0.96, 1.28)	1.00	1.14 (0.93, 1.39)	1.52 (0.98, 2.35)	1.42 (0.99, 2.04)	0.004
1+	38,118	1.10 (1.01, 1.19)	1.13 (1.05, 1.21)	1.00	1.08 (0.96, 1.21)	1.31 (1.00, 1.72)	1.40 (1.08, 1.81)	0.0008
Smoking								
Never	38,377	1.18 (1.09, 1.28)	1.17 (1.10, 1.26)	1.00	1.14 (1.02, 1.27)	1.55 (1.19, 2.01)	1.38 (1.07, 1.80)	<0.0001
Ever	7,944	1.05 (0.89, 1.25)	0.93 (0.80, 1.09)	1.00	0.97 (0.78, 1.21)	1.26 (0.83, 1.92)	1.77 (1.26, 2.48)	0.014
Alcohol								
Never	29,049	1.17 (1.07, 1.28)	1.17 (1.09, 1.27)	1.00	1.14 (1.01, 1.29)	1.44 (1.05, 1.98)	1.49 (1.10, 2.01)	<0.0001
Ever	17,194	1.14 (1.01, 1.28)	1.05 (0.94, 1.16)	1.00	1.05 (0.91, 1.23)	1.49 (1.10, 2.03)	1.60 (1.21, 2.11)	0.002
Never used tobacco or alcohol	27,891	1.18 (1.08, 1.30)	1.19 (1.10, 1.29)	1.00	1.15 (1.02, 1.31)	1.44 (1.04, 2.02)	1.35 (0.96, 1.88)	<0.0001
Coffee/caffeine containing soft drinks								
<Monthly	28,375	1.15 (1.05, 1.26)	1.16 (1.07, 1.25)	1.00	1.18 (1.04, 1.34)	1.59 (1.18, 2.15)	1.55 (1.17, 2.06)	<0.0001
Monthly or more often	15,787	1.18 (1.05, 1.34)	1.09 (0.98, 1.21)	1.00	0.96 (0.81, 1.13)	1.49 (1.06, 2.09)	1.66 (1.22, 2.26)	0.0005

Odds ratio (95 % CI). Adjusted for age when filling in the questionnaire and marital status

Of particulate interest in this population are associations in never smokers and women who have never used alcohol. Table 3 demonstrates that the relationships displayed in Table 2 were found in women who had abstained from smoking for their entire life and in women who had not, although the relationship may be somewhat stronger in the latter group (*p* value for interaction = 0.04). The association was the same in women who were lifelong abstainers from alcohol and other women (*p* value for interaction = 0.5) and was also found in the 27,891 women who denied ever having used either stimulant (alcohol and tobacco). Caffeine consumption was not an effect modifier. There was in addition a seemingly interaction for age at menarche, but the relationships for women with early and late menarche were the same (*p*-value for interaction = 0.9) if the two categories of obesity were merged.

The same stratified analyses were conducted also for irregular periods and miscarriages, and the relationships displayed in Table 2 were found consistently in the different strata of the population. For irregular periods, there were no indications of any significant interactions. For miscarriages, we found a weak U-formed (*p* = 0.003) relationship in parous women which was unaffected by further adjustments for parity. We refer to web appendix (Web tables 1–3) for more detailed presentation of stratified analyses.

## Discussion

Obesity at age 20 years was in this large study of women associated with increased risk of irregular periods and failing to become pregnant even if trying for one straight year, but not with the risk of experiencing at least one miscarriage.

As we are exploring relationships between body mass index at age 20 and reproductive problems, we have restricted the analytical sample to women at an age (aged 40 and above) when they most likely will have experienced reproductive problems if they will ever do so, particularly if these problems should have clinical consequences. As detailed above, we did not include irregular periods or failing be become pregnant before the age of 20 as an outcome in the study, only problems after the age of 20. We do not, however, know when the women experienced her (first) miscarriage; this may have happened as a teenager. Due to the mean age of the included women (nearly 60 years), modern treatment for infertility (like in vitro fertilization) has played a minor role for our findings.

The relationship between BMI at age 20 and irregular periods and problems of becoming pregnant may be explained by an increased risk of oligo- and anovulation in obese women and a number of other adverse effects of obesity on reproductive physiology in women [1–3, 25]. Our results support previous findings of a U-formed relationship between body mass index at age 23 and problems of becoming pregnant and menstrual problems before age 33. Even obesity at age 7 may be a predictor for the latter [18].

Data from a self-administered questionnaire hamper the possibilities for further discussion with regard to etiology. One explanation may be that a relatively high percentage of the obese women may have had polycystic ovary syndrome (PCOS). Polycystic ovary syndrome affects 5–10 % of women in a general population, and many (at least one out of three) of the patients with PCOS are obese [26, 27].

No relationship was found between obesity and the odds of reporting one or more miscarriages. Previous population-based studies have not given a consistent picture with regard to body mass index as a risk factor for miscarriages; underweight may be just as important as obesity [6–12]. Traditionally, PSOS has been thought to play a major role also for the risk of recurrent miscarriages [28], but this has recently been questioned [29].

The lack of relationship between body mass at age 20 and miscarriages may to some extent be due to misclassification as early miscarriages often are overlooked and any relationship will tend to be attenuated. However, the positive, direct relationship between the number of miscarriages and the odds of reporting failing to become pregnant indicates that information about the miscarriages has some validity and may further suggest that some women have interpreted the question “Did you ever try for one straight year or more to become pregnant and, during that time, not become pregnant?” less precisely than was the intention, answering that they for one straight year or more were not able to get pregnant *or give birth to a live born child.*

Given that there is no, or only a very weak, relationship between obesity and the risk of a miscarriage and that some women have interpreted the question as suggested above, this points to a possible stronger relationship between obesity (and possible underweight) and failure to become pregnant than the results presented in Table 2 may indicate.

Whereas irregular periods and failing to become pregnant reflect problems of becoming pregnant, the women who have a miscarriage have conceived and are therefore fertile. Thus, according to our findings, weight may be more important for becoming pregnant than for remaining pregnant. When the relationship with failing to become pregnant was adjusted for irregular periods and miscarriages, the odds ratio associated with obesity was attenuated. However, it is debatable whether it is correct to adjust for these variables which most likely are on the causal pathway. If not, obesity (BMI ≥ 30 kg/m^2^) increases the odds of having problems of becoming pregnant with approximately 45 %, even after adjustments for other likely confounders.

Table 1 shows that women who were obese at age 20 were less likely to ever have been married. We adjusted for marital status in all analyses (Table 2) and the stratified analyses by ever married status (Table 3) clearly demonstrate that the relationship we found is not due to women who never were married and therefore may not have tried to become pregnant. One might assume that experiencing irregular periods, the variable most strongly related to obesity, was independent of marital status, but the risk was found to be higher in never married women. We have, however, adjusted all relationships for marital status.

Our study has some limitations. There is a positive relationship between BMI in spouses [15, 30]. Obese men have reduced fertility [1, 31]. Thus, the higher odds for reporting problems of becoming pregnant in obese women may be due to obesity in the male partner. We are not able to link spouses in our database. It is however unlikely that the positive (and stronger) association (Table 2) between obesity and irregular periods is related to male obesity.

The information from the women did not make it possible to differentiate between a miscarriage (a spontaneous loss of a fetus before the 20th week of pregnancy) and a stillbirth (a delivery after 20 completed weeks’ gestation of a fetus showing no signs of life) [32]. The former is much more frequent, and our results will pertain largely to miscarriages. Wilcox et al. [5] found that 31 % of pregnancies were lost, two out of three before the pregnancy was detected clinically. Currently <1 % of all pregnancies in the US end as a stillbirth, but the percentage is higher in blacks than in other ethnic groups [32]. The risk of a stillbirth was higher during the childbearing years of the women included in our analysis, though. Obesity has in most studies been found to increase the risk of stillbirths [32, 33].

The women were asked to state their current height. We have used this height when computing the body mass earlier in life, at age 20. Thus, our analyses have most likely somewhat overestimated the BMI at age 20. However, as the associations we found were basically independent of age at enrollment (aged 40–54, 55–69, or ≥70, and thus time since the women were 20 years old), little bias is introduced when applying current height when computing BMI earlier in life.

The main weakness of our study is that weight is self-reported and recalled. Underweight women tend to overestimate the self-reported weight whereas obese women underestimate it [34]. Thus, in women with BMI < 18.5 kg/m^2^, the reported BMI is probably higher than the true BMI and the opposite is true for obese women. However, the most important in our context is the ability to rank the women according to BMI and measured and self-reported BMI has been found to be highly correlated (r_s_ = 0.94) in this population [35] as in the previous Adventist Health Study (AHS-1) [36].

The mean age at enrolment was 59.9 years, and the women were asked to recall their weight nearly 40 years earlier, at age 20. Data from the Nurses’ Health Study [37] indicate that women are able to recall their weight at age 18, the correlation coefficient between recalled and measured weight was 0.87. The women in the NHS cohort (aged 25–42) were, however, significantly younger than in our study. Data from women who took part in both this Adventist Health Study (AHS-2) and the former one (AHS-1 in 1976) demonstrate strong correlations (r = 0.82 for women of all ages) between recalled weight in the 1970s and weight stated in the questionnaires in 1976 [38]. Thus, misclassification of recalled BMI at least in terms of relative rank appears to be quite small for recall of 25–30 years.

We do not find it likely that our results can be explained by differential recall of weight at age 20 as this would imply a strong correlation between reporting problems of becoming pregnant or, in particular, irregular periods and falsely recalled overweight and obesity when aged 20 years old.

We only have data concerning weight and height and thus BMI. It is probable that a more relevant measure is the percentage of body fat, a measure that was strongly correlated (r = 0.84) with BMI in US women aged 20–39 [39]. Information about adipose tissue distribution, like waist circumference or waist/hip-ratio, may have given additional information, although the correlation between BMI and waist circumference in relatively young women is high (r = 0.93) according to recent NHANES data [39].

One possible source of bias would be that women who complete the lifestyle questionnaire are survivors. Obese, relatively young, women have higher mortality than women with normal weight [40, 41]. However, the mortality in women aged less than 40 is low, particularly in this relatively healthy group of subjects with low smoking prevalence, and the relationships did not depend on the age of the women when completing the questionnaire (Table 3 and web tables 1–3). Thus, it is unlikely that survival bias has impacted on our results to any measurable degree.

The prevalence of obesity at age 20 (2.4 %) is relatively low, but it is for instance similar to the prevalence of obesity in women included in the SWAN cohort which was based on women aged 17–18 years old in the late 1960s [42], Furthermore, the study population is somewhat selected as all the women were Adventists. It could be that obesity is associated with irregular periods and miscarriages differently in this group of women than in the general population. However, we find this unlikely and the stratified analyses did not suggest any interaction with lifestyle.

The study has, however, significant strengths. It is large in terms of women included, which has allowed detailed stratified analyses. The main findings were found to be very consistent in the different strata of the population. Another related strength is that this study has been conducted in a rather unique US population with a relatively high proportion of women who have abstained from alcohol and smoking for their entire life.

Additionally, 25 % of the analytical population are black Adventists of US and Caribbean origin; approximately 90 % of the remaining 75 % are white, non-Hispanic women. Both underweight and obesity was associated with being black (Table 1). After adjustment for age and marital status and compared to other women, black women were only slightly more likely to report irregular periods (2 %) or failing to become pregnant even if trying for least 1 year (6 %). However, blacks were more likely to report at least one miscarriage [OR = 1.34 (95 % CI: 1.27–1.40)]. However, as detailed in Table 3 and the web appendix, stratified analyses demonstrate that there are few indications that ethnicity has influenced our findings significantly.

In summary, this large study found that women who were obese when they were 20 years old were at a significantly increased risk of failing to become pregnant even if trying for one straight year. One of the explanations for this seems to be that obese women have difficulties to conceive due to irregular periods, rather than increased risk of miscarriages.

## Electronic supplementary material

Below is the link to the electronic supplementary material.

Supplementary material 1 (DOCX 36 kb)

## Abbreviations

CI: Confidence interval
BMI: Body mass index
