# Supplementary material for: Obesity at age 20 and the risk of miscarriages, irregular periods and reported problems of becoming pregnant: the Adventist Health Study-2
Source: Eur J Epidemiol. 2012 Dec 8;27(12):923–31. doi: 10.1007/s10654-012-9749-8 (PMC3539069; doi:10.1007/s10654-012-9749-8)
Supplement: Supplementary file 1 — Supplementary material 1 (DOCX 36 kb) [file 10654_2012_9749_MOESM1_ESM.docx]

Web table 1. Stratified analyses of the relationships between body mass index at age 20 and having experienced a miscarriage. Odds ratio (95 % CI). Adjusted for age when filling in the questionnaire and marital status.

|  |  | Body mass index (kg/m^2^) at age 20 | | | | | |  |
| --- | --- | --- | --- | --- | --- | --- | --- | --- |
|  | N | < 18.5 | 18.5-19.9 | 20-24.9 | 25-29.9 | 30-32.4 | ≥ 32.5 | *P* value |
| All women | 46334 | 1.06 (1.00, 1.12) | 1.02 (0.97, 1.07) | 1.00 | 1.11 (1.03, 1.20) | 1.11 (0.92, 1.35) | 0.97 (0.80, 1.16) | 0.06 |
| Age (years) : 40-54 | 18318 | 0.98 (0.89, 1.08) | 0.98 (0.90, 1.06) | 1.00 | 1.04 (0.92, 1.18) | 0.93 (0.70, 1.25) | 0.91 (0.71, 1.18) | 0.88 |
| 55-69 | 16670 | 1.13 (1.03, 1.24) | 1.07 (0.99, 1.17) | 1.00 | 1.21 (1.05, 1.38) | 1.60 (1.15, 2.21) | 1.04 (0.76, 1.43) | 0.002 |
| 70 + | 11346 | 1.10 (0.97, 1.25) | 1.01 (0.91, 1.12) | 1.00 | 1.09 (0.94, 1.27) | 0.85 (0.53, 1.36) | 0.94 (0.56, 1.57) | 0.57 |
| p-value for interaction |  | | | | | | | 0.86 |
| Never been married | 2615 | 0.99 (0.69, 1.44) | 1.03 (0.73, 1.44) | 1.00 | 1.18 (0.82, 1.71) | 0.55 (0.20, 1.53) | 0.46 (0.20, 1.06) | 0.32 |
| Ever been married | 43719 | 1.06 (1.00, 1.13) | 1.02 (0.97, 1.07) | 1.00 | 1.10 (1.02, 1.20) | 1.15 (0.94, 1.41) | 1.02 (0.84, 1.23) | 0.07 |
| p-value for interaction |  | | | | | | | 0.41 |
| Blacks | 11452 | 1.03 (0.92, 1.14) | 1.00 (0.90, 1.11) | 1.00 | 1.05 (0.91, 1.21) | 1.02 (0.73, 1.43) | 0.82 (0.60, 1.12) | 0.81 |
| Other ethnic groups | 34371 | 1.04 (0.97, 1.12) | 1.02 (0.96, 1.08) | 1.00 | 1.12 (1.02, 1.23) | 1.15 (0.90, 1.47) | 1.05 (0.83, 1.33) | 0.23 |
| p-value for interaction |  | | | | | | | 0.90 |
| Without college degree | 30901 | 1.05 (0.98, 1.13) | 1.04 (0.97, 1.10) | 1.00 | 1.18 (1.07, 1.29) | 1.14 (0.91, 1.41) | 0.98 (0.80, 1.21) | 0.016 |
| With college degree | 15037 | 1.08 (0.97, 1.19) | 1.00 (0.91, 1.09) | 1.00 | 0.92 (0.78, 1.07) | 0.91 (0.58, 1.42) | 0.83 (0.55, 1.25) | 0.47 |
| p-value for interaction |  | | | | | | | 0.03 |
| Nulliparous women | 7461 | 0.96 (0.81, 1.13) | 1.04 (0.90, 1.20) | 1.00 | 0.87 (0.70, 1.08) | 1.15 (0.72, 1.82) | 0.67 (0.44, 1.03) | 0.30 |
| Parous women | 38873 | 1.08 (1.01, 1.15) | 1.02 (0.96, 1.08) | 1.00 | 1.17 (1.07, 1.27) | 1.14 (0.92, 1.42) | 1.13 (0.91, 1.39) | 0.0028 |
| p-value for interaction |  | | | | | | | 0.0038 |
| Web table 1 continues… |  | | | | | | |  |
| Early (< 13 years) menarche | 23717 | 1.07 (0.97, 1.17) | 1.00 (0.93, 1.07) | 1.00 | 1.14 (1.03, 1.26) | 1.22 (0.96, 1.56) | 1.00 (0.81, 1.24) | 0.07 |
| Late (13 + years) menarche | 22317 | 1.07 (0.99, 1.16) | 1.04 (0.97, 1.12) | 1.00 | 1.06 (0.94, 1.20) | 0.96 (0.69, 1.34) | 0.85 (0.58, 1.25) | 0.46 |
| p-value for interaction |  | | | | | | | 0.66 |
| Never menstrual irregularities | 38329 | 1.06 (0.99, 1.13) | 1.03 (0.98, 1.09) | 1.00 | 1.12 (1.03, 1.22) | 1.20 (0.96, 1.51) | 0.93 (0.74, 1.16) | 0.05 |
| Ever menstrual irregularities | 6284 | 1.12 (0.95, 1.31) | 1.01 (0.88, 1.16) | 1.00 | 1.08 (0.88, 1.32) | 0.87 (0.58, 1.33) | 1.05 (0.73, 1.51) | 0.72 |
| p-value for interaction |  | | | | | | | 0.73 |
| Never problems getting pregnant | 37907 | 1.08 (1.01, 1.16) | 1.03 (0.97, 1.09) | 1.00 | 1.10 (1.01, 1.21) | 1.16 (0.93, 1.45) | 0.94 (0.76, 1.17) | 0.05 |
| Ever problems getting pregnant | 7529 | 0.94 (0.82, 1.08) | 0.97 (0.86, 1.09) | 1.00 | 1.13 (0.94, 1.35) | 0.86 (0.57, 1.31) | 0.91 (0.62, 1.33) | 0.56 |
| p-value for interaction |  | | | | | | | 0.57 |
| Not extended use of OC^1^ | 44989 | 1.07 (1.01, 1.14) | 1.03 (0.97, 1.08) | 1.00 | 1.10 (1.02, 1.19) | 1.11 (0.91, 1.36) | 0.98 (0.81, 1.18) | 0.06 |
| Extended use of OC^1^ | 866 | 0.60 (0.36, 0.98) | 0.83 (0.55, 1.26) | 1.00 | 1.08 (0.58, 2.02) | 1.80 (0.52, 6.31) | 0.32 (0.04, 2.73) | 0.23 |
| p-value for interaction |  | | | | | | | 0.20 |
| Never smoked | 38194 | 1.04 (0.98, 1.11) | 1.00 (0.95, 1.06) | 1.00 | 1.11 (1.02, 1.21) | 1.07 (0.84, 1.35) | 0.89 (0.70, 1.12) | 0.14 |
| Ever smoked | 7868 | 1.14 (0.99, 1.31) | 1.10 (0.98, 1.24) | 1.00 | 1.10 (0.92, 1.30) | 1.15 (0.80, 1.64) | 1.06 (0.78, 1.46) | 0.38 |
| p-value for interaction |  | | | | | | | 0.65 |
| Never used alcohol | 28957 | 1.08 (1.00, 1.16) | 1.02 (0.95, 1.08) | 1.00 | 1.11 (1.00, 1.23) | 1.08 (0.81, 1.43) | 0.97 (0.73, 1.27) | 0.22 |
| Ever used alcohol | 17023 | 1.04 (0.95, 1.15) | 1.02 (0.94, 1.11) | 1.00 | 1.09 (0.97, 1.23) | 1.13 (0.86, 1.49) | 0.95 (0.74, 1.23) | 0.66 |
| p-value for interaction |  | | | | | | | 0.99 |
| Never used tobacco or alcohol | 27796 | 1.05 (0.98, 1.14) | 1.00 (0.93, 1.06) | 1.00 | 1.12 (1.01, 1.24) | 1.08 (0.80, 1.45) | 1.00 (0.74, 1.33) | 0.28 |
| Do not drink coffee/caffeine containing soft drinks | 28170 | 1.03 (0.95, 1.11) | 0.99 (0.93, 1.06) | 1.00 | 1.07 (0.96, 1.18) | 1.18 (0.91, 1.54) | 0.88 (0.68, 1.13) | 0.42 |
| Drinks coffee/caffeine containing soft drinks | 15674 | 1.09 (0.98, 1.21) | 1.04 (0.96, 1.14) | 1.00 | 1.12 (0.98, 1.28) | 0.99 (0.72, 1.36) | 1.13 (0.85, 1.51) | 0.38 |
| p-value for interaction |  | | | | | | | 0.61 |

^1^ Used oral contraceptives (OC) for 7 or more years both when aged 20-29 and when aged 30-39.

Web table 2.Stratified analyses of the relationships between body mass index at age 20 and having experienced menstrual irregularities. Odds ratio (95 % CI). Adjusted for age when filling in the questionnaire and marital status.

|  |  | Body mass index (kg/m^2^) at age 20 | | | | | |  |
| --- | --- | --- | --- | --- | --- | --- | --- | --- |
|  | N | < 18.5 | 18.5-19.9 | 20-24.9 | 25-29.9 | 30-32.4 | ≥ 32.5 | *P* value |
| All women | 45701 | 1.03 (0.95, 1.12) | 0.98 (0.91, 1.04) | 1.00 | 1.18 (1.07, 1.31) | 1.89 (1.53, 2.33) | 1.98 (1.64, 2.39) | < 0.0001 |
| Age (years) : 40-54 | 18426 | 1.07 (0.96, 1.20) | 1.03 (0.94, 1.14) | 1.00 | 1.28 (1.12, 1.46) | 2.16 (1.65, 2.83) | 2.04 (1.61, 2.58) | < 0.0001 |
| 55-69 | 16498 | 1.02 (0.89, 1.17) | 0.90 (0.79, 1.01) | 1.00 | 1.00 (0.82, 1.21) | 1.73 (1.17, 2.56) | 2.06 (1.45, 2.91) | < 0.0001 |
| 70 + | 10777 | 0.92 (0.73, 1.16) | 0.97 (0.81, 1.16) | 1.00 | 1.26 (0.98, 1.61) | 1.14 (0.55, 2.39) | 1.36 (0.62, 3.00) | 0.40 |
| p-value for interaction |  | | | | | | | 0.96 |
| Never been married | 2590 | 0.80 (0.58, 1.10) | 1.15 (0.88, 1.50) | 1.00 | 1.17 (0.87, 1.58) | 2.09 (1.21, 3.64) | 2.02 (1.32, 3.08) | 0.0007 |
| Ever been married | 43111 | 1.05 (0.97, 1.14) | 0.96 (0.90, 1.04) | 1.00 | 1.18 (1.06, 1.31) | 1.86 (1.48, 2.34) | 1.96 (1.59, 2.42) | < 0.0001 |
| p-value for interaction |  | | | | | | | 0.36 |
| Blacks | 11299 | 0.99 (0.86, 1.14) | 0.95 (0.83, 1.09) | 1.00 | 1.04 (0.87, 1.25) | 1.75 (1.22, 2.52) | 2.11 (1.56, 2.87) | < 0.0001 |
| Other ethnic groups | 33914 | 1.04 (0.95, 1.15) | 0.98 (0.90, 1.06) | 1.00 | 1.25 (1.11, 1.41) | 1.99 (1.53, 2.58) | 1.91 (1.50, 2.42) | < 0.0001 |
| p-value for interaction |  | | | | | | | 0.63 |
| Without college degree | 30257 | 0.95 (0.86, 1.05) | 0.92 (0.85, 1.01) | 1.00 | 1.17 (1.03, 1.31) | 1.76 (1.37, 2.25) | 1.84 (1.47, 2.30) | < 0.0001 |
| With college degree | 15086 | 1.20 (1.05, 1.37) | 1.08 (0.96, 1.21) | 1.00 | 1.15 (0.95, 1.38) | 2.25 (1.49, 3.39) | 2.15 (1.49, 3.11) | < 0.0001 |
| p-value for interaction |  | | | | | | | 0.06 |
| Nulliparous women | 7163 | 0.95 (0.79, 1.14) | 0.92 (0.79, 1.08) | 1.00 | 1.15 (0.94, 1.41) | 1.60 (1.07, 2.39) | 1.87 (1.38, 2.54) | 0.0001 |
| Parous women | 37450 | 1.08 (0.98, 1.18) | 0.99 (0.92, 1.08) | 1.00 | 1.17 (1.04, 1.32) | 1.95 (1.52, 2.50) | 1.89 (1.49, 2.41) | < 0.0001 |
| p-value for interaction |  | | | | | | | 0.86 |
| Web table 2 continues… | | | | | | | | |
| Early (< 13 years) menarche | 23542 | 1.07 (0.95, 1.20) | 0.90 (0.81, 0.99) | 1.00 | 1.18 (1.04, 1.35) | 1.97 (1.52, 2.55) | 1.93 (1.55, 2.40) | < 0.0001 |
| Late (13 + years) menarche | 22031 | 1.00 (0.90, 1.12) | 1.04 (0.95, 1.15) | 1.00 | 1.19 (1.01, 1.40) | 1.69 (1.17, 2.45) | 2.07 (1.43, 3.00) | < 0.0001 |
| p-value for interaction |  | | | | | | | 0.24 |
| Never problems getting pregnant | 37921 | 1.02 (0.93, 1.12) | 0.97 (0.89, 1.05) | 1.00 | 1.15 (1.02, 1.29) | 1.93 (1.52, 2.46) | 2.01 (1.62, 2.49) | < 0.0001 |
| Ever problems getting pregnant | 7419 | 1.04 (0.88, 1.22) | 0.96 (0.83, 1.12) | 1.00 | 1.24 (1.00, 1.54) | 1.64 (1.06, 2.55) | 1.74 (1.17, 2.59) | 0.0066 |
| p-value for interaction |  | | | | | | | 0.93 |
| Never miscarriage | 30997 | 1.03 (0.93, 1.13) | 0.99 (0.91, 1.08) | 1.00 | 1.20 (1.06, 1.35) | 2.03 (1.58, 2.61) | 1.91 (1.53, 2.39) | < 0.0001 |
| Ever miscarriage | 13616 | 1.11 (0.97, 1.28) | 0.96 (0.85, 1.09) | 1.00 | 1.15 (0.96, 1.38) | 1.58 (1.06, 2.36) | 2.25 (1.57, 3.21) | < 0.0001 |
| p-value for interaction |  | | | | | | | 0.70 |
| Not extended use of OC^1^ | 44373 | 1.04 (0.96, 1.12) | 0.97 (0.90, 1.04) | 1.00 | 1.19 (1.08, 1.32) | 1.91 (1.54, 2.36) | 1.96 (1.62, 2.37) | < 0.0001 |
| Extended use of OC^1^ | 876 | 1.09 (0.66, 1.78) | 1.01 (0.64, 1.58) | 1.00 | 0.54 (0.22, 1.31) | 1.22 (0.31, 4.78) | 2.02 (0.57, 7.15) | 0.63 |
| p-value for interaction |  | | | | | | | 0.57 |
| Never smoked | 37662 | 1.03 (0.95, 1.13) | 1.01 (0.93, 1.09) | 1.00 | 1.22 (1.09, 1.37) | 1.86 (1.44, 2.39) | 2.12 (1.69, 2.67) | < 0.0001 |
| Ever smoked | 7786 | 1.06 (0.88, 1.26) | 0.85 (0.73, 1.00) | 1.00 | 1.02 (0.82, 1.27) | 1.75 (1.18, 2.60) | 1.61 (1.15, 2.25) | 0.0005 |
| p-value for interaction |  | | | | | | | 0.30 |
| Never used alcohol | 28430 | 1.04 (0.94, 1.15) | 0.96 (0.87, 1.05) | 1.00 | 1.23 (1.08, 1.41) | 1.94 (1.43, 2.64) | 1.76 (1.32, 2.35) | < 0.0001 |
| Ever used alcohol | 16943 | 1.04 (0.92, 1.18) | 1.01 (0.91, 1.12) | 1.00 | 1.12 (0.97, 1.30) | 1.82 (1.36, 2.44) | 2.13 (1.66, 2.74) | < 0.0001 |
| p-value for interaction |  | | | | | | | 0.73 |
| Never used tobacco or alcohol | 27313 | 1.04 (0.93, 1.15) | 0.95 (0.87, 1.05) | 1.00 | 1.25 (1.09, 1.44) | 1.89 (1.37, 2.60) | 1.75 (1.29, 2.39) | < 0.0001 |
| Do not drink coffee/caffeine containing soft drinks | 27866 | 1.02 (0.93, 1.13) | 0.90 (0.82, 0.99) | 1.00 | 1.05 (0.91, 1.20) | 1.82 (1.36, 2.44) | 1.85 (1.43, 2.39) | < 0.0001 |
| Drinks coffee/caffeine containing soft drinks | 15595 | 1.04 (0.90, 1.19) | 1.08 (0.97, 1.21) | 1.00 | 1.32 (1.13, 1.54) | 2.06 (1.50, 2.82) | 2.11 (1.57, 2.82) | < 0.0001 |
| p-value for interaction |  | | | | | | | 0.07 |

^1^ Used oral contraceptives (OC) for 7 or more years both when aged 20-29 and when aged 30-39.

Web table 3. Stratified analyses of the relationships between body mass index at age 20 and having experienced problems getting pregnant. Odds ratio (95 % CI). Adjusted for age when filling in the questionnaire and marital status.

|  |  | Body mass index (kg/m^2^) at age 20 | | | | | |  |
| --- | --- | --- | --- | --- | --- | --- | --- | --- |
|  | N | < 18.5 | 18.5-19.9 | 20-24.9 | 25-29.9 | 30-32.4 | ≥ 32.5 | *P* value |
| All women | 46582 | 1.16 (1.08, 1.25) | 1.13 (1.06, 1.20) | 1.00 | 1.11 (1.01, 1.22) | 1.46 (1.17, 1.82) | 1.55 (1.26, 1.90) | < 0.0001 |
| Age (years) : 40-54 | 18514 | 1.21 (1.09, 1.35) | 1.08 (0.98, 1.19) | 1.00 | 1.23 (1.07, 1.42) | 1.56 (1.15, 2.12) | 1.55 (1.18, 2.04) | < 0.0001 |
| 55-69 | 16795 | 1.11 (0.98, 1.25) | 1.10 (1.00, 1.23) | 1.00 | 0.95 (0.79, 1.13) | 1.42 (0.96, 2.10) | 1.60 (1.12, 2.28) | 0.014 |
| 70 + | 11273 | 1.15 (0.97, 1.35) | 1.28 (1.13, 1.46) | 1.00 | 1.10 (0.90, 1.34) | 1.26 (0.73, 2.17) | 1.31 (0.72, 2.41) | 0.008 |
| p-value for interaction |  | | | | | | | 0.92 |
| Never been married | 2607 | 1.31 (0.81, 2.12) | 0.76 (0.45, 1.30) | 1.00 | 1.27 (0.77, 2.09) | 1.49 (0.58, 3.85) | 1.64 (0.81, 3.31) | 0.33 |
| Ever been married | 43975 | 1.16 (1.08, 1.25) | 1.14 (1.07, 1.21) | 1.00 | 1.10 (1.00, 1.21) | 1.45 (1.16, 1.83) | 1.53 (1.23, 1.89) | < 0.0001 |
| p-value for interaction |  | | | | | | | 0.59 |
| Blacks | 11583 | 1.12 (0.97, 1.28) | 0.97 (0.85, 1.10) | 1.00 | 1.22 (1.03, 1.46) | 1.43 (0.97, 2.11) | 1.29 (0.90, 1.85) | 0.031 |
| Other ethnic groups | 34489 | 1.17 (1.07, 1.27) | 1.19 (1.11, 1.27) | 1.00 | 1.07 (0.95, 1.20) | 1.47 (1.12, 1.94) | 1.69 (1.31, 2.17) | < 0.0001 |
| p-value for interaction |  | | | | | | | 0.028 |
| Without college degree | 31013 | 1.18 (1.08, 1.29) | 1.14 (1.06, 1.24) | 1.00 | 1.08 (0.97, 1.22) | 1.60 (1.25, 2.05) | 1.65 (1.31, 2.07) | < 0.0001 |
| With college degree | 15181 | 1.14 (1.01, 1.29) | 1.09 (0.98, 1.21) | 1.00 | 1.15 (0.96, 1.38) | 1.06 (0.63, 1.77) | 1.26 (0.80, 1.98) | 0.21 |
| p-value for interaction |  | | | | | | | 0.38 |
| Nulliparous women | 7318 | 1.33 (1.13, 1.55) | 1.11 (0.96, 1.28) | 1.00 | 1.14 (0.93, 1.39) | 1.52 (0.98, 2.35) | 1.42 (0.99, 2.04) | 0.004 |
| Parous women | 38118 | 1.10 (1.01, 1.19) | 1.13 (1.05, 1.21) | 1.00 | 1.08 (0.96, 1.21) | 1.31 (1.00, 1.72) | 1.40 (1.08, 1.81) | 0.0008 |
| p-value for interaction |  | | | | | | | 0.47 |
| Web table 3 continues… | | | | | | | | |
| Early (< 13 years) menarche | 23903 | 1.17 (1.05, 1.30) | 1.16 (1.06, 1.27) | 1.00 | 1.12 (0.99, 1.27) | 1.15 (0.86, 1.54) | 1.74 (1.38, 2.19) | < 0.0001 |
| Late (13 + years) menarche | 22498 | 1.16 (1.06, 1.28) | 1.11 (1.02, 1.21) | 1.00 | 1.07 (0.92, 1.25) | 2.08 (1.47, 2.93) | 0.92 (0.57, 1.49) | < 0.0001 |
| p-value for interaction |  | | | | | | | 0.024 |
| Never menstrual irregularities | 38972 | 1.15 (1.06, 1.25) | 1.14 (1.06, 1.22) | 1.00 | 1.05 (0.94, 1.18) | 1.47 (1.12, 1.91) | 1.48 (1.15, 1.91) | < 0.0001 |
| Ever menstrual irregularities | 6368 | 1.15 (0.97, 1.37) | 1.11 (0.95, 1.29) | 1.00 | 1.17 (0.94, 1.45) | 1.25 (0.81, 1.90) | 1.33 (0.91, 1.95) | 0.27 |
| p-value for interaction |  | | | | | | | 0.91 |
| Never miscarriage | 31550 | 1.22 (1.11, 1.33) | 1.15 (1.06, 1.25) | 1.00 | 1.11 (0.98, 1.26) | 1.58 (1.19, 2.09) | 1.58 (1.22, 2.04) | < 0.0001 |
| Ever miscarriage | 13886 | 1.06 (0.94, 1.20) | 1.10 (0.99, 1.22) | 1.00 | 1.12 (0.96, 1.31) | 1.15 (0.79, 1.70) | 1.50 (1.06, 2.14) | 0.11 |
| p-value for interaction |  | | | | | | | 0.56 |
| Not extended use of OC^1^ | 45212 | 1.17 (1.09, 1.26) | 1.13 (1.07, 1.21) | 1.00 | 1.11 (1.01, 1.23) | 1.45 (1.15, 1.81) | 1.50 (1.22, 1.85) | < 0.0001 |
| Extended use of OC^1^ | 876 | 0.75 (0.37, 1.50) | 0.96 (0.54, 1.68) | 1.00 | 0.74 (0.25, 2.16) | 2.95 (0.72, 12.1) | 4.23 (1.02, 17.6) | 0.18 |
| p-value for interaction |  | | | | | | | 0.27 |
| Never smoked | 38377 | 1.18 (1.09, 1.28) | 1.17 (1.10, 1.26) | 1.00 | 1.14 (1.02, 1.27) | 1.55 (1.19, 2.01) | 1.38 (1.07, 1.80) | < 0.0001 |
| Ever smoked | 7944 | 1.05 (0.89, 1.25) | 0.93 (0.80, 1.09) | 1.00 | 0.97 (0.78, 1.21) | 1.26 (0.83, 1.92) | 1.77 (1.26, 2.48) | 0.014 |
| p-value for interaction |  | | | | | | | 0.04 |
| Never used alcohol | 29049 | 1.17 (1.07, 1.28) | 1.17 (1.09, 1.27) | 1.00 | 1.14 (1.01, 1.29) | 1.44 (1.05, 1.98) | 1.49 (1.10, 2.01) | < 0.0001 |
| Ever used alcohol | 17194 | 1.14 (1.01, 1.28) | 1.05 (0.94, 1.16) | 1.00 | 1.05 (0.91, 1.23) | 1.49 (1.10, 2.03) | 1.60 (1.21, 2.11) | 0.0016 |
| p-value for interaction |  | | | | | | | 0.53 |
| Never used tobacco or alcohol | 27891 | 1.18 (1.08, 1.30) | 1.19 (1.10, 1.29) | 1.00 | 1.15 (1.02, 1.31) | 1.44 (1.04, 2.02) | 1.35 (0.96, 1.88) | < 0.0001 |
| Do not drink coffee/caffeine containing soft drinks | 28375 | 1.15 (1.05, 1.26) | 1.16 (1.07, 1.25) | 1.00 | 1.18 (1.04, 1.34) | 1.59 (1.18, 2.15) | 1.55 (1.17, 2.06) | < 0.0001 |
| Drinks coffee/caffeine containing soft drinks | 15787 | 1.18 (1.05, 1.34) | 1.09 (0.98, 1.21) | 1.00 | 0.96 (0.81, 1.13) | 1.49 (1.06, 2.09) | 1.66 (1.22, 2.26) | 0.0005 |
| p-value for interaction |  | | | | | | | 0.42 |

^1^ Used oral contraceptives (OC) for 7 or more years both when aged 20-29 and when aged 30-39.
